# Advances in Pediatric Urologic Laparoscopy

**DOI:** 10.1100/tsw.2007.141

**Published:** 2007-01-22

**Authors:** M. C. Smaldone, E. Polsky, D. J. Ricchiuti, S. G. Docimo

**Affiliations:** Division of Pediatric Urology, Children's Hospital of Pittsburgh, Department of Urology, University of Pittsburgh School of Medicine, Pittsburgh, Pennsylvania, USA

**Keywords:** laparoscopy, pediatrics, urology

## Abstract

The spectrum of laparoscopic surgery in children has undergone a dramatic evolution. Initially used as a diagnostic modality for many pediatric urologists, complex as well as reconstructive procedures are now being performed laparoscopically. Laparoscopic orchiopexy and nephrectomy are well established and are being performed at many centers. Laparoscopic partial nephrectomy, adrenalectomy, and dismembered pyeloplasty series have reported shortened hospital stays and operative times that are comparable to that of open techniques or are decreasing with experience. The initial experiences with laparoscopic ureteral reimplantation and laparoscopic-assisted bladder reconstructive surgery have been described, reporting encouraging results with regards to feasibility, hospital stay, and cosmetic outcome. This report will provide a directed review of the literature to establish the current indications for laparoscopy in pediatric urologic surgery.

## INTRODUCTION

Pediatric urological laparoscopy is evolving rapidly. Potential benefits over traditional techniques include smaller incisions, increased magnification improving visualization, reduced postoperative pain, shorter postoperative ileus, fewer wound complications, and shorter hospital stays. However, it has been difficult to show differences in results compared to open procedures, which historically have been excellent for children. Due to longer operating times and increased hospital costs without a measurable difference in recovery time for younger patients, laparoscopy has been slower to catch on for children than for the adult population[1], and complex procedures are only done in a few specialized centers. However, over the past decade, there has been a steady increase in the application of laparoscopic techniques that have evolved from simple diagnostic maneuvers to complex reconstructive procedures. This report aims to provide a directed review of the literature to establish the current indications for laparoscopy in pediatric urologic surgery.

## ANESTHESIA

The anatomical and physiological differences in children include high chest wall compliance, diaphragmatic respiration, and rate-dependent cardiac output due to reduced ventricular compliance, resulting in a decreased respiratory compliance and increased airway pressures with the creation of a pneumoperitoneum[2]. Halachmi et al.[3] retrospectively reviewed 62 patients undergoing laparoscopic procedures and compared the impact of extraperitoneal vs. intraperitoneal carbon dioxide insufflation on several cardiopulmonary variables. They reported that both intraperitoneal and extraperitoneal insufflation resulted in significant increases in respiratory rate, peak airway pressures, and end-tidal carbon dioxide with minor changes in heart rate and oxygen saturation[3]. While the safety of laparoscopic procedures has been documented in complicated pediatric cases[4], the physiological effects of carbon dioxide insufflation are important to consider in children with comorbid cardiopulmonary compromise. El Ghoneimi et al.[5] demonstrated the feasibility of laparoscopic retroperitoneal nephrectomy in a high-risk cohort of children with end-stage renal disease prior to transplantation. Although there were measured elevations of systolic blood pressure and end-tidal carbon dioxide, no additional treatment was required to correct these physiologic changes, and there were no intraoperative complications[5]. In addition, it should be noted that, despite concerns to the contrary, there is no documented increase in risk of pneumoperitoneum in children with ventriculoperitoneal shunts[6].

## COMPLICATIONS

Since the introduction of diagnostic laparoscopy for abdominal exploration of undescended testes, the indications for laparoscopy in pediatric urology have expanded rapidly. Contemporary series of adult laparoscopic procedures report complication rates ranging from 12–16%[7,8]. However, there are few such studies examining the laparoscopic complications in the pediatric population. In 1996, Peters[9] reviewed 5400 laparoscopic cases performed by 153 pediatric urologists, reporting a complication rate of 5.4%. However, excluding misdirected insufflation, the incidence of complications decreased to 1.2%, of which 0.4% required surgical repair. Peters also concluded that the greatest predictor of complication rate was laparoscopic experience[9]. In 2003, Esposito et al.[10] reviewed 701 laparoscopic procedures performed in eight institutions, reporting 19 (2.7%) complications, of which six required conversion to open surgery. Interestingly, surgical team experience in this review was not related to complication rate. These studies reaffirm that laparoscopic procedures in children are safe, with minimal mortality and low rate of complications.

It is important to note that as the level of complexity of attempted laparoscopic procedures increases, the incidence of complications may rise above these reported figures. Initially, in the mid 1990s, the few pediatric urologists performing these procedures had a great deal of laparoscopic experience. Today, the breadth of urologists attempting laparoscopic procedures has expanded and, likewise, the level of resident involvement has increased as well. However, to address the need for expansion of resident education, Farhat et al.[11] demonstrated that mentored laparoscopic teaching is a safe way to introduce advanced urological laparoscopic procedures to inexperienced residents or physicians.

## MANAGEMENT OF NONPALPABLE TESTES

### Diagnostic Laparoscopy for Cryptorchidism

Of the 1% of males diagnosed with cryptorchidism[12], as many as 20% will have a nonpalpable testis[13]. Historically, if cord vessels were not visualized during inguinal exploration, laparotomy was the standard procedure to search for the testis or blind-ending vessels and establish the diagnosis of a vanishing testis[14]. Radiologic testing has limited value in detection or localization of nonpalpable testicles[15,16,17], which are most commonly peeping, intra-abdominal, or atrophic (vanishing). Initially described by Cortesi et al. in 1976[18], diagnostic laparoscopy has become the gold standard for the evaluation and treatment of the nonpalpable testicle.

Recent studies report that blind-ending cord structures or an intra-abdominal testis are found during laparoscopic evaluation of nonpalpable testis between 31–83% of the time[19,20,21,22,23,24,25]. Barqawi et al.[22] reviewed 27 patients who had undergone previous inguinal exploration and identified a viable canalicular or intra-abdominal testicle in 67%. Cisek et al.[26] reported that laparoscopic findings precluded unnecessary abdominal exploration in 13% of cases and that the typical surgical incision for inguinal exploration would have left the surgeon compromised in 66% of the cases compared to the approach optimized as a result of laparoscopic testicular localization. In many of these patients, diagnostic laparoscopy can eliminate the need for further open exploration, or facilitate open or laparoscopic orchiopexy.

Alternative strategies in the evaluation of nonpalpable testes have been described. To reduce the number of unnecessary intra-abdominal laparoscopic procedures, Kanemoto et al.[27] suggested initial inguinal exploration followed by transinguinal laparoscopy. In 22 patients with a hypertrophied unilateral palpable testis, Belman et al.[28] reported that scrotal exploration prior to laparoscopic evaluation identified an atrophic testicular remnant in 91% of patients. Schleef et al.[29] described a technique in which they proceeded first with laparoscopic evaluation of the inguinal canal to avoid unnecessary open exploration. The decision to proceed with an inguinal or laparoscopic abdominal exploration first depends on the surgeon’s certainty on physical exam and is still currently a source for debate. However, the evidence clearly suggests that if an initial open inguinal exploration is inconclusive, laparoscopic exploration should be the next step in the diagnosis and treatment of the nonpalpable testis. Our current protocol is to perform examination under anesthesia. If any tissue suggestive of a scrotal nubbin is felt, then open scrotal exploration is performed. If this exam is negative or inconclusive, only then do we perform laparoscopic exploration.

## Laparoscopic Orchiopexy

Laparoscopic techniques have been applied to the therapy of intra-abdominal testes as an extension of diagnosis. The impetus for development of these techniques has been the difficulty of achieving successful open orchiopexy for the high undescended testis. In a meta-analysis of open orchiopexy techniques, Docimo[30] reported success rates by type of procedure (inguinal, 89%; Fowler-Stephens, 67%; staged Fowler-Stephens, 77%; transabdominal, 81%; two-stage, 73%; microvascular, 84%) and concluded that the high failure rates left significant room for improvement. There have been many technical descriptions of laparoscopic orchiopexy with similar success rates; our approach has been previously described[31]. Lindgren et al.[32] reported a 93% success rate in the treatment of 44 nonpalpable testes in 36 patients with no evidence of testicular atrophy. Initial laparoscopic orchiopexy series, although viewed as promising, were criticized for originating from high-volume centers with increased laparoscopic experience and low patient numbers[32,33,34,35,36,37,38]. To address this issue, in a large multi-institutional analysis, Baker et al.[39] reported excellent success rates superior to that of historical open orchiopexy, and no significant difference in success or complication rates between low- and high-volume centers.

The decision to perform a single-stage procedure leaving the vessels intact, or to perform a one- or two-stage Fowler-Stephens procedure, is challenging and no specific set of criteria has been determined. Baker et al.[39] reported the incidence of testicular atrophy after primary laparoscopic (2.2%), one-stage Fowler-Stephens (22%), and two-stage Fowler-Stephens (10%) orchiopexy. Some centers advocate universal two-stage procedures, whereas others try to be more selective. Intra-operatively measuring the distance between the testis and the internal ring, observation of the cord anatomy, or the ability of the intra-abdominal testicle to reach the opposite inguinal ring can be helpful[40]. Laparoscopy has also been used as an adjunct to microvascular testicular autotransplantation[41]. Compared to open orchiopexy, the early data show that laparoscopic orchiopexy is a successful approach with low risk in the management of the nonpalpable undescended testicle.

## RENAL SURGERY

The laparoscopic nephrectomy has become the standard of care in the adult population and has been embraced by pediatric urologists as well. The first laparoscopic nephrectomy was performed in a child by Kavoussi and Koyle in 1992[42]. Subsequent reports have demonstrated the efficacy of laparoscopic nephrectomy, partial nephrectomy, heminephrectomy, and nephroureterectomy in children for a variety of etiologies.

### Laparoscopic Nephrectomy

Laparoscopic nephrectomies in the pediatric population are most commonly performed for nonfunctional kidneys secondary to obstructive uropathy, reflux, or ectopic ureteral implantation. Although initially described through a transperitoneal approach, the retroperitoneal approach is also used[43]. This is a matter of personal choice, as no specific advantage has been proven for either. A retroperitoneal approach through prone positioning has been described as well, with excellent results[44].

Initial series described the laparoscopic nephrectomy as effective and safe, with a low incidence of open conversion[43,45,46,47]. El Ghoneimi et al.[43] described their initial experience with 42 retroperitoneal laparoscopic renal procedures in 41 children in 1998. In the 31 patients undergoing nephrectomy, mean operative time was 104 min, mean hospital length of stay was 2 days, and two patients required conversion to an open procedure due to unidentified polar vessels[43]. Hamilton et al.[48] retrospectively compared 20 patients undergoing open and laparoscopic nephrectomies for benign disease, and reported a significantly shorter hospital stay in the laparoscopic group (22.5 vs. 41.3 h), with no significant difference in operative time. El-Ghoneimi et al.[5] retrospectively compared open and laparoscopic nephrectomy approaches in 18 high-risk children with end-stage renal disease prior to transplantation. They reported no intraoperative complications or conversion to open procedures, and similarly reported a shorter mean hospital stay in the laparoscopic group, with no significant difference in mean operative length. While current data are encouraging and supportive of the widespread use of laparoscopy in renal surgery, a prospective study comparing nephrectomies via an open vs. laparoscopic approach has yet to be performed.

### Laparoscopic Partial Nephrectomy, Heminephrectomy, and Nephroureterectomy

The primary indication for a partial or heminephrectomy in a pediatric patient is to remove a nonfunctioning upper or lower pole due to a duplex collecting system anomaly. Compared to adults in which a partial nephrectomy is typically performed for malignancy, a laparoscopic approach in children is more straightforward due to clear anatomic and vascular planes between the upper and lower duplex systems, decreasing the risk for damage to the vascular supply to the remnant pole[49]. Initially described via a transperitoneal approach in a 14-year-old girl in 1993 by Jordan and Winslow[50], this procedure has been subsequently performed through lateral and prone retroperitoneal approaches as well (Table 1). The retroperitoneal approach allows posterior access to the renal pedicle without entering the peritoneal cavity, decreasing the need to mobilize the conserved moiety or the dissection of its blood supply. Borzi and Yeung[51] suggest that a posterior retroperitoneal approach is preferred for isolated renal or polar excision without extended ureterectomy, while a lateral retroperitoneal approach provides better exposure to ectopically located kidneys and the distal ureter. They speculated that the transperitoneal approach should be reserved for cases when complete moiety excision with lower urinary tract reconstruction is anticipated[51]. We perform these procedures exclusively transperitoneally with excellent exposure and results.

**TABLE 1**
**Laparoscopic Partial/Heminephrectomy**

| Study | Number of Patients/ Procedures | Approach (TP/RP)* | Mean Operative Time (min) | Mean Hospital Stay (days) | Conversion to Open | Complications |
|---|---|---|---|---|---|---|
| Janetschek et al.[52]** | 14/17 | TP | 222 (UP) 427 (LP) | 4.4 (UP) 7.5 (LP) | 0 | 0 |
| Horowitz et al.[53] | 13/14 | TP | 100 | 2.6 | 0 | 1 decrease in hematocrit managed conservatively |
| El-Ghoneimi, et al.[57] | 15 | RP | 152 | 1.4 | 1 | 1 urinoma |
| Valla et al.[54] | 24 | RP | 160 | 3.4 | 3 (12.5%) | 9 (37%) intraoperative; 5 (20%) postoperative urinomas |
| Robinson et al.[58] | 11 | TP | 200.4 | 25.5 h | 1 | 1 ureteral triplication requiring reoperation |
| Lee et al.[59] | 14 | RP | 194 | 1.7 | 0 | 1 urinoma |
| Piaggio et al.[60] | 14 | TP | 180 | 2 | 0 | 2 (omental hernia, urinoma) |

* TP, transperitoneal; RP, retroperitoneal.

** 12 UP (upper pole), 5 LP (lower pole) heminephrectomies.

In their initial series of 14 children undergoing upper- or lower-pole laparoscopic partial nephrectomies, Janetchek et al.[52] reported that the procedure can be performed with minimal blood loss and low morbidity, despite an increased operative time. In a series of 14 transperitoneal upper-pole partial nephrectomies, Horowitz et al.[53] reported that laparoscopic techniques provided better field magnification, minimal blood loss, faster recovery, and less surgical scarring than historical open controls. In a series of 24 pediatric partial nephrectomies using the retroperitoneal approach, Valla et al.[54] reviewed their intraoperative (37%) and postoperative (20%) complication rates. Of the nine intraoperative complications, five consisted of peritoneal tears requiring no treatment and four injuries (16%), including a duodenal injury requiring open conversion and two lower-pole calyceal injuries repaired laparoscopically, required intraoperative intervention[54]. In an outcome analysis of retroperitoneal laparoscopic heminephrectomies in 23 children, Wallis et al.[55] reported conversion to an open procedure in four patients (17%), a urine leak in four patients (17%), and functional loss of the remaining ipsilateral renal moiety in two children (9%). It is important to note that both renal functional losses occurred in patients less than 1 year of age, leading some authors to speculate that a transperitoneal laparoscopic or even open approach may be more appropriate in this age group[56]. In a comparison between 15 laparoscopic and 13 open partial nephrectomies, El-Ghoneimi et al.[57] reported no differences in intraoperative blood loss (<20 ml) and operative duration (152 vs. 146 min). However, those in the laparoscopic group had a significantly shorter length of hospital stay (1.4 vs. 3.9 days)[57]. Robinson et al.[58] compared 22 partial nephrectomies performed by either a retroperitoneal laparoscopic or open approach, and reported decreased length of hospital stay and lower analgesic requirements in the laparoscopic group. However, they also reported an increased cost ($6,125 vs. $4,244) and a significant difference in mean operative time (200.4 vs. 113.5 min) in the laparoscopic group. Lee et al.[59] performed a case-control retrospective study comparing an age-matched cohort of 28 pediatric patients undergoing open partial nephrectomy to those undergoing laparoscopic partial nephrectomy. The laparoscopic cohort had comparable mean operative times (193 vs. 194 min), a decreased mean hospital stay (1.7 vs. 4.7 days), and less postoperative analgesia (0.44 vs. 1.53 mg/kg) without conversion or significant complications[59]. More recently, Piaggio et al.[60] reported low complication rates (one omental hernia and one urinoma) in 14 young children undergoing laparoscopic partial nephrectomy, a rate comparable to 20 similarly aged patients undergoing open partial nephrectomy. Both the retroperitoneal and transperitoneal approaches have been shown to be efficacious and safe with diminished postoperative morbidity, even in young children, with mean operative times approaching that of traditional open results.

## Laparoscopic Pyeloplasty

Open pyeloplasty has long been the standard of care in the management of UPJ obstruction. Laparoscopic pyeloplasty was introduced in adult patients in 1993[61] and in children by Peters et al. in 1995[62]. Laparoscopic pyeloplasty in children has followed a similar evolution as other forms of laparoscopic renal surgery. Individual surgeons have preference for transperitoneal or retroperitoneal approaches. The retroperitoneal approach has been criticized for increased operating times secondary to operating in a limited working space, making suturing more difficult. It has been stated that the transperitoneal approach increases the risk of abdominal visceral injury and adhesion formation, but there is no evidence to support this[63].

Tan[64] reported the first transperitoneal laparoscopic dismembered pyeloplasty series of 18 children in 1999. Mean laparoscopic time was 89 min and no patient required conversion to an open procedure, although two patients required repeat laparoscopic pyeloplasty for persistent obstruction. Other transperitoneal pyeloplasty series (Table 2) have shown comparable efficacy and success rates[64,65,66,67,68]. In a series of 46 children, Metzelder et al.[68] reported that the laparoscopic transabdominal pyeloplasty was safe and effective in all age groups (0–1, 1–7, and 7–18 years). In a series of eight infants aged 3–5 months, Kutikov et al.[67] demonstrated that a transperitoneal approach was safe and effective in children less than 6 months of age. In the largest series to date, our group[69] performed 49 laparoscopic Anderson-Hynes pyeloplasties, two laparoscopic Heineke-Mikulicz pyeloplasties, and one laparoscopic pyeloureterostomy in 52 children (mean age of 10.7 years) and reported a median hospital stay of 1 day, no intraoperative complications or open conversion, and a 96% success rate.

Yeung et al.[70] reported the first series of retroperitoneal laparoscopic dismembered pyeloplasties in 13 patients, in which the mean laparoscopic time was 143 min and one required conversion to an open procedure. In a series of 22 retroperitoneal laparoscopic pyeloplasties, El Ghoneimi et al.[71] reported a mean operative time of 228 min with a mean hospital stay of 2.5 days. In four cases, conversion to open surgery was necessary. Other smaller series have shown similar efficacy rates[63,72]. In the only retrospective comparison between laparoscopic and open pyeloplasties published to date, Bonnard et al.[63] reported a significant decrease in both hospital stay (2.4 vs. 5 days) and postoperative acetaminophen requirement for patients undergoing retroperitoneal laparoscopic pyeloplasties, but a significantly longer mean operative time (219 vs. 96 min). The advantages of the laparoscopic approach appear to be a shorter hospital stay and decreased analgesia requirement postoperatively, as well as a markedly improved cosmetic result. Operative times decrease with greater laparoscopic experience and, in our series, are approaching times for open pyeloplasty.

**TABLE 2**
**Laparoscopic Pyeloplasty**

| Study | Number of Patients/ Procedures | Approach (TP/RP)* | Mean Laparoscopic Time (min) | Mean Hospital Stay (days) | Conversion to Open |
|---|---|---|---|---|---|
| Tan et al.[64] | 18/18 | TP | 89 | 2 | 0 |
| Yeung et al.[70] | 13/13 | RP | 143 | NA | 1 (8%) |
| El-Ghoneimi et al.[71] | 21/22 | RP | 228 | 2.5 | 4 (18%) |
| Farhat et al.[72] | 11/12 | RP | 160 | 2 | 2 (17%) |
| Casale et al.[65] | 19/19 | TP | 186 | 3 | 0 |
| Reddy et al.[66] | 16/16 | TP | 160 | NA | 0 |
| Bonnard et al.[63]** | 22/22 | RP | 219 | 2.4 | 0 |
| Kutikov et al.[67]*** | 8/8 | TP | 108 | 1.2 | 0 |
| Metzelder et al.[68] | 46/46 | TP | 175 | NA | 2 (4%) |
| Sweeney et al.[69] | 52/52 | TP | 296† | 1.5 | 0 |

* TP, transperitoneal; RP, retroperitoneal.

** Two patients requiring open conversion were excluded from the study.

*** All eight patients were between the ages of 3–5 months at the time of procedure.

† Represents total operating room time including induction, cystoscopy, and repositioning.

## ADRENAL SURGERY

### Laparoscopic Adrenalectomy

Initially described in the early 1990s, laparoscopic adrenalectomy is currently recognized as the gold standard in the treatment of benign adrenal lesions in the adult population. With technical advances and increased experience, surgeons have successfully performed laparoscopic adrenalectomies for metastatic and primary malignancies of the adrenal gland as well. Over the past 5 years, the advantages of the laparoscopic management of adrenal lesions have been applied to the pediatric population as well.

Initial series have described retroperitoneal and transperitoneal laparoscopic approaches, and have reported that these procedures can be performed safely and effectively with a short hospital stay, minimal blood loss, and excellent functional outcome. In a series of six children, Mirallie et al.[73] performed six transperitoneal and two retroperitoneal laparoscopic adrenalectomies for adrenal masses with a mean tumor size of 4 cm. Conversions to open procedures were necessary for left adrenal glands in two children. In 13 children with adrenal lesions (mean size 4.1 cm), Castilho et al.[74] reported a mean operative time of 107 min and a mean hospital stay of 5.5 days. There were no reported intraoperative complications, and two of the 13 procedures (15.4%) were converted to open surgery[74]. In a series of 22 laparoscopic adrenalectomies in 21 children, Skarsgard et al.[75] reported a mean operative time of 101 min and a mean postoperative hospital stay of 1.5 days, with conversion to open adrenalectomy in one patient with a left adrenal carcinoma and tumor thrombus extending into the renal vein. In 17 children with mean 4.8-cm adrenal lesions, Miller et al.[76] reported a mean operating time of 120 min, mean estimated blood loss of 25 ml, and mean length of hospitalization of 35 h. Other small series have demonstrated the efficacy of laparoscopy in partial adrenalectomies[73], local tumor recurrence resection after prior open adrenalectomy[77], and treatment of neuroblastoma[78]. In a retrospective comparison of 60 children undergoing open adrenalectomy and four children undergoing laparoscopic adrenalectomy for benign disease, Stanford et al.[79] reported equivalent total hospital costs, mean blood loss, and mean operative times, with significant differences in hospital length of stay (5.4 vs. 2.7 days). Despite their small size, the results from these initial series of laparoscopic adrenalectomies in pediatric patients are comparable to the adult literature, and show that in selected patient populations at medical centers with advanced laparoscopic experience, nearly all types of adrenal tumors can be removed by laparoscopy.

## RECONSTRUCTIVE LOWER URINARY TRACT SURGERY

### Ureteral Reimplantation

Traditional open ureterovesical reimplantation procedures have been extremely successful with success rates of 95–98% in pediatric populations. A minimally invasive approach with extravesical ureteral reimplantation was first introduced in pig models[80,81,82]. This was followed by two small clinical series of two and six patients, respectively, in the early 1990s[83,84]. While speculating that the advantages of a laparoscopic approach would lead to shorter length of hospital stay, less morbidity, and better cosmesis, the authors felt that the technical demands of the procedure and longer mean operative times outweighed the benefits compared to established open techniques.

In a series of 71 reimplants in 47 children (23 unilateral and 24 bilateral), Lakshmanan et al.[85] modified the Lich-Gregoir laparoscopic extravesical approach and reported a 100% success rate with no persistent reflux or obstruction. They recommended a careful selection of operative candidates, speculating that ureters with ureteroceles and megaureters that need tapering are unsuitable, and that the working space in the pelvis of children younger than 4 years may be inadequate for laparoscopic techniques.

More recently, a pneumovesicoscopic approach has been introduced[86]. The bladder is filled with carbon dioxide to improve visibility. This has been used clinically, and the concept was proven feasible in animal studies[87]. In 29 children (46 refluxing ureters), Gatti et al.[88] performed percutaneous endoscopic trigonoplasty (Gil-Vernet technique in 23 patients, Cohen technique in six patients). Although they reported higher success rates with the Cohen technique (83 vs. 63%), mean operative times were longer and overall success rates were significantly lower than traditional open reimplants[88]. Okamura et al.[89] reported similar success rates (70%) in 15 children undergoing endoscopic trigonoplasty. Overall results for this technique have not been satisfactory when compared to traditional open reimplant success rates, causing this approach to fall out of favor.

In a more recent iteration of this approach, the laparoscope is placed through a transabdominal approach instead of per urethra, which had previously limited mobility[90]. Yeung et al.[91] reported their initial experience with this technique in 16 children (seven bilateral, 23 refluxing ureters), reporting a mean operative time of 136 min (112 min unilateral, 178 min bilateral), and an overall success rate of 96% with one conversion to an open procedure who had displacement of a port into the extravesical space. Although there have been limited studies exploring this technique's benefits compared to open surgery, it is unlikely that this more complex approach will replace an operation done through a small bikini incision with high success, unless some specific benefit is proven.

### Laparoscopic-Assisted Reconstructive Surgery

Pediatric patients with a neurogenic bowel or bladder occasionally require reconstructive surgery to improve quality of life, self-confidence, and become more independent[92]. Historically, the creation of continent urinary stomas and antegrade continence enemas (ACE) has been accomplished as an open procedure, utilizing a midline incision to isolate the bowel segment for harvesting. Initially described in a case report of appendicovesicostomy by Jordan and Winslow in 1993[93], Hedican et al.[94] reported the first series of eight patients (mean age 13.4 years) undergoing a variety of laparoscopic-assisted reconstructive procedures (including bladder augmentation, appendiceal Mitrofanoff procedure, and tapered ileal Mitrofanoff and Malone ACE). They concluded that laparoscopic-assisted surgery allows for mobilization of bowel segments with reconstruction via a low midline or Pfannenstiel incision, allowing for a more rapid recovery and an improved cosmetic appearance[94].

Cadeddu et al.[95] compared 11 cases of laparoscopic-assisted ACE and/or continent urinary stoma creation with a cohort of five standard open cases performed during the same period. They reported similar operative lengths (6.1 vs. 6.2 h) with significant differences in resumption of regular diet (2.3 vs. 5.6 days) and mean hospital stay (4.1 vs. 7.4 days), with minimal complications[95]. Ureterocystoplasty with associated nephrectomy can be accomplished through a cosmetically acceptable Pfannenstiel incision, with reduced morbidity and an acceptable operative duration as well[96]. In the largest series to date, our group[97] reviewed 31 patients undergoing laparoscopic-assisted reconstructive surgery through a lower midline or Pfannenstiel incision for a variety of etiologies. They reported that only one case required conversion to an open procedure due to adhesions, and at mean follow up of 19 months; 95% of 39 continent stomas in 29 patients were continent and easily catheterizable. Small series and case reports have been published describing pure laparoscopic gastrocystoplasty[98] and enterocystoplasty[99], but at this juncture, the technical demands of these procedures are not generally acceptable. However, with increasing experience, laparoscopic-assisted reconstructive surgery is being performed by a growing group of pediatric urologists. It is our strong feeling that long midline incisions are obsolete for this group of children.

## INGUINAL SURGERY

### Laparoscopic Varicocelectomy

The reported incidence of varicoceles in the pediatric or adolescent population is between 10–15%, some of which have been shown to affect ipsilateral testicular growth[100,101,102]. Because it is impossible to predict which adolescents with varicoceles will have related fertility issues at this time, the major indication for repair has been ipsilateral testicular hypertrophy. It has been documented that catch-up growth of a smaller testis can occur in greater than 80% after successful treatment[103]. Traditionally, the most popular treatment for varicoceles in childhood has been the open high retroperitoneal ligation of the vasa spermatica interna (Palomo procedure), resulting in a low incidence of varicocele recurrence, but a high postoperative hydrocele rate[104,105]. However, in the early 1990s, initial series concluded that the laparoscopic approach to varicocele correction is safe and effective, with similar results to the open technique[106,107,108].

Several studies have compared the laparoscopic varicocelectomy repair with open techniques in adult populations[109,110]. Although there is considerable variation between individual series, the majority reported longer mean operative times and increased costs, with similar efficacy, shorter hospital stays, and improved cosmetic results. With recent technical advances including the single trocar technique (compared to as many as four trocar sites described initially), recent series have reported a decrease in operative times with increasing laparoscopic experience[109,111]. These results have been duplicated in the pediatric population. In a randomized controlled trial comparing 434 children undergoing laparoscopic varicocelectomy and 220 children undergoing open varicocelectomy, Podkamenev et al.[112] reported lower rates of hydroceles, wound complications, and testicular or scrotal edema, with shorter mean operative times, hospital length of stay, and postoperative analgesia use in the laparoscopic group. In a series of 122 children and adolescents, Koyle et al.[113] reported a mean operative time of 28 min (with a mean operative time of 14.6 min in the most recent 28 cases), catch-up growth in 64 of 78 testicles at 1 year (82%), and hydroceles in six patients. Riccabona et al.[114] recently compared four operative techniques (laparoscopic, inguinal artery sparing, standard Palomo, and modified Palomo) in 121 children undergoing 128 varicocelectomies. Comparison of the four groups revealed significant differences in varicocele recurrence and hydrocele formation. Of the 19 patients undergoing intraperitoneal laparoscopic varicocelectomy, there were three cases of hydrocele formation (two reactive, which spontaneously resolved), two patients with persistent or recurrent varicocele, and testes volume increased in 84%[114].

Recently, Hassan et al.[115] reported a 22.8% hydrocele rate after 79 laparoscopic varicocelectomies in children. Hydrocele formation has been reduced to 0–2% in some recent series incorporating microsurgical techniques[116,117]. With recent advances in operative technology, laparoscopic varicocele repairs are being performed with shorter operative times and decreased complication rates, resulting in lower hospital costs for the patients approaching that of the traditional open repair. Many of these benefits can be achieved using an open subinguinal approach, and each has its own proponents and technical challenges.

## ROBOTIC SURGERY

A recent technicological advance in the field of pediatric laparoscopic surgery is the application of robotic technology. Initially described by Partin et al.[118] in the adult urological population, few studies have examined its use in pediatric urology. Potential advantages of robotic-assisted laparoscopy include an enhanced ability to perform precise suturing for reconstruction, enhanced stereoscopic visualization offering true depth-of-field vision, and increased dexterity, while the chief deterrents are increased cost, lack of tactile feedback, and the lack of pediatric sized ports and instruments[119]. Initial experiences in performing continent diversion with appendix[120] and heminephrectomy[121] in children have been reported. Olsen et al.[86] have described pneumovesical ureteral reimplantation in a pig model, which has been expanded to include both extra- and intravesical approaches in children[122].

The delicate intracorporal suturing inherent to the laparoscopic pyeloplasty seems a natural candidate for robotic application to reduce the learning curve for performing laparoscopic procedures. In a comparison between laparoscopic pyeloplasty performed with the da Vinci (Intuitive Surgical, Sunnyvale, CA) robotic system matched to procedures performed with standard laparoscopic techniques, Gettman et al.[123] reported decreased operative times with robotic-assisted pyeloplasty compared with standard laparoscopic pyeloplasty. However, these results have not been duplicated. In a prospective comparison in adults, Link et al.[124] reported that mean operative and total room time for robotic laparoscopic pyeloplasty was significantly longer than standard laparoscopic pyeloplasty by 19.5 and 39.0 min, respectively. Further, the robotic laparoscopic pyeloplasty was 2.7 times more costly than the laparoscopic pyeloplasty due to longer operative time, increased consumables costs, and depreciation of the da Vinci system. Link et al. concluded that for the experienced laparoscopist, application of the da Vinci robot resulted in no significant clinical advantage and added substantial cost to transperitoneal laparoscopic dismembered pyeloplasty. Initial studies in children have demonstrated that although technically feasible and safe, operative times do not approach that of the standard open procedure, and there is no clear advantage in reduction of postoperative morbidity compared to the standard laparoscopic pyeloplasty[125,126,127,128]. However, early results with robotic-assisted laparoscopy are encouraging and warrant further evaluation in pediatric urologic surgery. It is the opinion of the authors that, as of this date, the robot is most helpful to those early in the learning curve and its major value will be in increasing access to minimally invasive procedures in centers lacking experience in complex laparoscopic techniques. Hopefully, the technology will advance to a point where it becomes advantageous to those with significant reconstructive experience.

## CONCLUSION

The spectrum of laparoscopic urological surgery in children continues to expand. Currently, procedures such as laparoscopic exploration for undescended testicles and laparoscopic nephrectomy have been accepted as gold standard and are performed at most institutions. Other procedures such as the laparoscopic pyeloplasty and laparoscopic reconstructive surgery have only recently been introduced and are primarily available at centers with advanced laparoscopic experience. The level of complexity of these cases will be associated with longer operative times until surgeons become more proficient. Both transperitoneal and retroperitoneal approaches are safe and effective. Robotic-assisted laparoscopic surgery has the potential to provide the advantages of the reduced morbidity of laparoscopy without sacrificing reconstructive precision, but it is expensive and of unproven benefit. It is our hope that minimally invasive approaches to the undescended testicle, UPJ obstruction, lower tract reconstruction, and other conditions will become more available to children in the near future.
